# Fourier Transform Infrared Imaging and Infrared Fiber Optic Probe Spectroscopy Identify Collagen Type in Connective Tissues

**DOI:** 10.1371/journal.pone.0064822

**Published:** 2013-05-22

**Authors:** Arash Hanifi, Helen McCarthy, Sally Roberts, Nancy Pleshko

**Affiliations:** 1 Tissue Imaging and Spectroscopy Laboratory, Department of Bioengineering, Temple University, Philadelphia, Pennsylvania, United States of America; 2 Robert Jones & Agnes Hunt Orthopaedic Hospital and ISTM, Keele University, Oswestry, Shropshire, United Kingdom; University of Rochester, United States of America

## Abstract

Hyaline cartilage and mechanically inferior fibrocartilage consisting of mixed collagen types are frequently found together in repairing articular cartilage. The present study seeks to develop methodology to identify collagen type and other tissue components using Fourier transform infrared (FTIR) spectral evaluation of matrix composition in combination with multivariate analyses. FTIR spectra of the primary molecular components of repair cartilage, types I and II collagen, and aggrecan, were used to develop multivariate spectral models for discrimination of the matrix components of the tissues of interest. Infrared imaging data were collected from bovine bone, tendon, normal cartilage, meniscus and human repair cartilage tissues, and composition predicted using partial least squares analyses. Histology and immunohistochemistry results were used as standards for validation. Infrared fiber optic probe spectral data were also obtained from meniscus (a tissue with mixed collagen types) to evaluate the potential of this method for identification of collagen type in a minimally-invasive clinical application. Concentration profiles of the tissue components obtained from multivariate analysis were in excellent agreement with histology and immunohistochemistry results. Bone and tendon showed a uniform distribution of predominantly type I collagen through the tissue. Normal cartilage showed a distribution of type II collagen and proteoglycan similar to the known composition, while in repair cartilage, the spectral distribution of both types I and II collagen were similar to that observed via immunohistochemistry. Using the probe, the outer and inner regions of the meniscus were shown to be primarily composed of type I and II collagen, respectively, in accordance with immunohistochemistry data. In summary, multivariate analysis of infrared spectra can indeed be used to differentiate collagen type I and type II, even in the presence of proteoglycan, in connective tissues, using both imaging and fiber optic methodology. This has great potential for clinical in situ applications for monitoring tissue repair.

## Introduction

Damage or degeneration of cartilage is frequently associated with changes in the macromolecular structure and content of the primary cartilage components [1], and can ultimately progress to osteoarthritis (OA) [2]. The progression of OA has been directly linked to the loss of proteoglycans (PGs) [3], and to changes in collagen structure and orientation [4]. It is widely accepted that cartilage injuries do not heal spontaneously [5], which is related to the avascular nature of the tissue and the limited ability of mature chondrocytes to proliferate and regenerate new cartilage [6]. Although there is no cure for OA, there have been advances in the treatment of cartilage focal defects. Methods such as microfracture and autologous chondrocyte implantation (ACI) can stimulate cells to make extracellular matrix (ECM) components, and at times, result in a spatial structure similar to normal cartilage [5], [7]. Studies have shown that different modalities result in different types of repair tissue. Microfracture generally results in fibrocartilage which contains type I collagen [3], whereas following ACI, a mixture of fibrocartilage and hyaline cartilage is generated [6]. The presence of hyaline cartilage, which is composed of type II collagen, is better correlated with a positive clinical outcome [8]–[10]. Type I and II collagen are among the fibril forming proteins with a triple helical secondary structure in the collagen family, however, there are some differences in their structure. The former is a heterotrimer of COL1A1 and COL1A2 genes products, and the latter is a homotrimer of COL2A1 gene products. Type I collagen is usually incorporated with collagen type III and V, while type II collagen is incorporated with type IX and XI collagens that increase tissue load bearing properties and limit the fibril diameter to smaller sizes compared to type I collagen. Type II collagen contains a greater amount of hydroxylysine and glucosyl and galactosyl residues than type I collagen, and these mediate the connection of collagen and proteoglycan components in the matrix [11]. Therefore, from a clinical perspective, knowledge of the collagen type present in the repair tissue is extremely important.

Currently, modalities to evaluate collagen quantity include enzymatic digestion of the tissue followed by biochemical hydroxyproline [12] or Sircol [13] assays. Evaluation of collagen type is typically performed by immunohistochemical (IHC) analysis on thin tissue sections, or by tissue digest separation methods such as high performance liquid chromatography (HPLC) followed by collagen type detection by mass spectroscopy (MS) [6], [14], [15]. These methods have different sensitivities to collagen amount and type, but all can be time consuming and require processing of the tissue. Fourier transform infrared (FT-IR) spectroscopy is a powerful technique based on molecular vibrations that has been widely used to assess biological tissue composition [3], [16]–[19]. FT-IR analysis relies on the unique vibrational signature of the molecules present in the tissue, and thus does not require the addition of an external contrast agent. [20]. FT-IR can be utilized as a clinical tool when coupled with an infrared fiber optic probe (IFOP) [21], [22], or can be used for analysis of harvested tissue when coupled with a microscope and an array detector [3], [16], [23], [24]. The latter modality, called FT-IR imaging spectroscopy (FT-IRIS), creates “chemical” images of tissues based on the absorbance of a specific molecular species at a pixel resolution as high as 1.5 µm, in combination with microscopic visualization of the samples [16]. FT-IRIS has been increasingly used to characterize the structure, distribution, and orientation of extracellular matrix molecules in histological sections of cartilage and other connective tissues [16], [25]–[27].

There is considerable overlap in the spectral signatures of proteins in connective tissues, and therefore, it is not always possible to identify a unique absorbance at a specific frequency that is associated with a particular component. Univariate analyses of IR spectra have been performed to evaluate collagen and proteoglycan (PG) concentration [3], [16], [23], [26]–[29]. Recent studies have shown that application of second derivative spectra in univariate analysis results in better measurement of matrix component [30]. However, multivariate analysis techniques that evaluate multiple frequencies of the spectra simultaneously are increasingly used to improve specificity of the analysis and predict matrix components quantitatively. Euclidean distance [31], [32], cluster analysis [24], and partial least squares (PLS) [21], [33], have all been used to analyze FT-IR spectra of cartilage. These studies demonstrated the ability to distinguish normal, repair, young and mature tissues, and to quantitatively analyze matrix components. To date, however, discrimination of regions of type II collagen in tissues with other collagen types by univariate or multivariate analysis of FT-IR spectra has not been demonstrated.

The aim of the current study was to assess whether FTIR spectroscopy, applied in both an imaging mode (to histological sections) and fiber optic modality (to the tissue surface), can differentiate collagen type I and II in connective tissues. A PLS model was developed based on a library of IR spectra of varying concentrations of pure tissue components including type I collagen, type II collagen, and aggrecan (the primary PG in cartilage). Assessment of the composition of tendon and bone (primarily type I collagen), hyaline cartilage (primarily type II collagen), repair cartilage (often a mix of types I and II collagen) and meniscus (known to consist of both type I and II collagen) was performed to validate the PLS model. In addition, multivariate cluster analysis was performed to evaluate an alternate method for discrimination of types I and II collagen in histological sections of tissues. Finally, the model was validated on fiber optic probe data acquired from an intact meniscus to demonstrate the potential of this method for in vivo clinical use.

## Materials and Methods

### Overview

Infrared spectra were collected from mixtures of varying amounts of pure type I and II collagen, and aggrecan to form a spectral library. The spectral library was used to predict the concentration of these components in histological sections of connective tissues using FT-IRIS, and on the surface of meniscus using IFOP assessment to show feasibility of this modality.

### Pure components

Type I collagen, type II collagen, and aggrecan powders were used to make mixtures that were analyzed as potassium bromide (KBr) pellets. Two different sets of samples were prepared that included mixtures of varying concentrations of type II collagen (chick nasal articular cartilage, Genzyme, Boston, MA) and aggrecan (calf nasal aggrecan, generously supplied by L. Rosenberg, Montefiore Hospital, Bronx, NY in a prior study [26]) (Model A), and mixtures of varying concentrations of type I collagen (bovine skin, USB Corporation, Cleveland, OH) and type II collagen (bovine articular cartilage, USB Corporation, Cleveland, OH) (Model B). Inclusion of type II collagen from two different sources permitted us to assess the sensitivity of the analysis to collagen source. Collagens and/or aggrecan were mixed as 1% by weight with 100 mg KBr powder (International Crystal Labs, Garfield, NJ) to create KBr pellets.

The composition of pellets for Model A (n = 10 pellets per mixture) and Model B (n = 3 pellets per mixture) are summarized in Table 1 and Table 2, respectively.

**Table 1:** The composition of pellets for Model A (n = 10 pellets per mixture).

Sample	1	2	3	4	5
**Type II collagen (wt %)**	0	25	50	75	100
**Aggrecan (wt %)**	100	75	50	25	0

**Table 2:** The composition of pellets for Model B (n = 3 pellets per mixture).

Sample	1	2	3	4	5
**Type I collagen (wt %)**	100	75	50	25	0
**Type II collagen (wt %)**	0	25	50	75	100

### Bovine tissue

Bovine knee joints (2–3 weeks old) were obtained from a local FDA approved abattoir (JBS Souderton, Souderton, PA). Cortical bone was harvested from the tibia and cut into 1×2×1 cm^3^ samples. The patellar tendon was harvested and cut into 2 cm^2^ pieces. Full depth cartilage, including the calcified cartilage layer, was harvested from the medial and lateral femoral condyles (5 mm diameter pieces). Lateral meniscus was harvested and cut in the sagittal plane for FT-IRIS, and used intact for infrared fiber optic probe (IFOP) spectroscopy. Bone and tendon were evaluated as standards for tissues considered to have very little or no type II collagen, while the articular cartilage matrix was considered a standard for a tissue with very little or no type I collagen. Meniscus was assessed as a model of a tissue that contains both type I and II collagen in its matrix. All tissues were fixed in 10% buffered formalin (Richard-Allan Scientific, Kalamazoo, MI) for 24 hours.

Bone and cartilage samples were decalcified in a 12.5% EDTA solution. After formalin fixation, tissues were put into 70% ethanol for dehydration prior to paraffin embedding. Paraffin blocks were sectioned at 6 and 7 micron thickness on glass slides and low-emissivity slides (low-e, Kevley Technologies, Chesterland, OH) for histology and FT-IRIS, respectively. Tendons were sectioned longitudinally, and meniscus and articular cartilage sectioned sagittally.

### Human tissue

Full-depth core biopsy samples (1.8 mm in diameter) were obtained from patients who had undergone autologous chondrocyte implantation (ACI) for a medial femoral condyle defect ∼12 months previously (N = 4). All tissues were obtained under approval of the NRES Research Ethics Committee (West Midlands, UK). Participants provided their written informed consent to participate in this study and the ethics committee approved this consent procedure. The tissues were immediately snap frozen and stored in liquid nitrogen until processed. Seven micron thick sections were cut and collected for analyses as above.

### Histological and immunohistochemical (IHC) analysis

Histologic sections of bone, tendon, meniscus and cartilage were stained using hematoxylin & eosin (H&E) and Alcian blue (for PG assessment) (Sigma-Aldrich, St. Louis, MO). Since both types I and type II collagen are generally present in repair cartilage, IHC images of the tissues were used as the gold standard for comparison. IHC staining was performed on cryosections of ACI biopsies using antibodies specific to type I and II collagen. Separate tissue sections were incubated for 1 hour at room temperature with primary antibodies against type I collagen (monoclonal antihuman, clone no 1-8H5; ICN) and type II collagen (CIICI; Developmental Studies Hybridoma Bank, IA) [6]. Endogenous peroxidase was blocked with 0.3% hydrogen peroxide in methanol before sections were incubated with the biotinylated secondary anti-mouse antibody. The signal was amplified using an avidin–biotin–peroxidase reagent (Vectastain Elite ABC kit; Vector Laboratories, Peterborough, UK) and labeling was visualized with diaminobenzidine as substrate. Sections were then washed, dehydrated, and mounted in pertex. ‘Control’ sections of biopsy samples (to assess nonspecific antibody binding) were treated either with normal mouse IgG, normal rabbit serum, or phosphate buffered saline alone, in place of the primary antibodies, and then treatment continued.

In addition, IHC staining of paraffin-embedded meniscus was performed for type I collagen using a primary antibody against type I collagen (anti-collagen type I, fetal mouse skin, Calbiochem, Merck Milipore, Darmstadt, Germany).

### FT-IR data acquisition

KBr pellets: FT-IR spectra from the Model A type II collagen and aggrecan KBr pellets had been previously collected as described in Camacho et al [26]. FT-IR spectra from the Model B type I and type II collagen KBr pellets were collected in the mid-IR region of 1800–900 cm^−1^ at 4 cm^−1^ spectral resolution using a Spectrum 400 FT-IR spectrometer (Perkin Elmer, Shelton, CT). The average time for data collection from each pellet was ∼2 minutes.

FT-IRIS data collection: FT-IRIS data were acquired from this sections of human and bovine tissues (n = 3 per tissue type) in the mid-IR region, 1800–750 cm^−1^ at 4 cm^−1^ spectral resolution and 25 µm spatial resolution using a Spectrum SpotLight 400 FT-IR Imaging system (Perkin Elmer, Shelton, CT). Depending on the size of the tissue section (0.5–1.5 mm^2^), data collection time ranged from ∼5–20 minutes.

IFOP data collection: To validate the ability to discriminate type I and type II collagen in intact tissues, infrared fiber optic probe (IFOP) data were collected from bovine meniscus in the mid-IR region of 1800-900 cm^−1^ at 4 cm^−1^ spectral resolution with 256 co-added scans (average time of scan for each spectrum: ∼1 minute), using a Bruker infrared spectrometer (Billerica, MA) equipped with a mercury cadmium telluride (MCT) detector and coupled to a chalcogenide fiber optic probe of ∼6 mm diameter (Remspec Corp, Charlton, MA) with a flat-tipped ZnS attenuated total reflectance (ATR) crystal of 1 mm diameter. The 1 mm tip of the probe was placed in direct contact at 90° to 24 individual regions on the meniscus femoral surface for data acquisition. Penetration of infrared radiation in IFOP data collection is restricted to the tissue surface to a depth of ∼10 microns [34]. Prior to multivariate analyses (described below), the spectral regions of the amide I protein absorbance (1718–1594 cm^−1^), the amide II protein absorbance (1594-1492 cm^−1^), and the PG sugar ring absorbance (1140-985 cm^−1^) were investigated to qualitatively assess component distribution in IR images [16].

### Multivariate analysis (PLS)

FT-IR spectra obtained from KBr pellets of pure component mixtures in the 1800-900 cm^−1^ range were used to create PLS models to validate that the spectra from these mixtures could indeed be differentiated based on concentration of components. This spectral range was chosen for analysis to correspond with the spectral range in the IFOP data. PLS analysis was originally developed based on regression analysis and principal component analysis (PCA), where the scores calculated by projection of the response and independent variables to a new space of principal components are used to find a linear regression model and predict unknown variables. PCA is a multivariate analysis method which is used to reveal hidden structure within data and provide visual representation of the relationship between variables and response. It decomposes the information carried by the original variables and projects them onto a smaller number of latent variables called principal components (PCs). In PLS analysis, a model is found that correlates PCs of the matrix of variables to PCs of the response using linear regression analysis [35]–[38]. A multiplicative scatter correction (MSC), second derivative differentiation with 13-point Savitzky–Golay smoothing, and normalization to the maximum peak height of all spectra were applied to optimize the models. MSC is a signal processing method which is used to remove the scattering and offset effects in the spectra [36]. IR spectra were employed as variables with each spectral frequency input as an x variable, and the actual type I collagen, type II collagen or PG content used as the response variables. All spectral variables (frequencies) were input with equal weighting. A leave-one-out cross validation was used to create the PLS models. In this method of validation, a repeated analysis is done on the data, where each time one of the samples is left out and the PLS model is optimized based on the remaining spectra, The model is then used on the left out sample for validation [38], [39]. The number of factors for the model was calculated to be seven, based on the minimal residual sum of squares error (SS_err_) [35].

Based on a leverage versus residual scatter plot of the PLS model [35], no outliers were detected in the PLS cross-validation models. The quality of the model was evaluated by assessment of the root mean square error (RMSE), and the regression coefficient (R^2^) of the cross validation model. RMSE measures the precision of the model and is calculated according to the equation below:
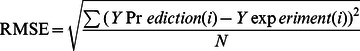
(1)(i: number of samples = 1 to N) [40], [41].

Once the PLS model was developed from the pure component KBr pellet data, the model was applied to the infrared spectra collected from different regions of the connective tissues of interest to predict composition. The number of FT-IRIS spectra collected spanned the length of the tissue section analyzed so that, e.g., 100 spectra collected at 25 micron pixel resolution = 2500 microns total length. The IR spectra included 100 FT-IRIS spectra of cortical bone, 100 FT-IRIS spectra of tendon (transverse direction), 80 FT-IRIS spectra of articular cartilage (from superficial zone to deep zone), 100 FT-IRIS spectra of meniscus (from the tip of inner region towards the outer region), and 24 IFOP spectra (1 mm region per spectrum) from the meniscus surface, from the inner to outer region. The output for the PLS model prediction is either percent dry weight of a specific component (for the dehydrated tissue sections), or percent wet weight (for the in situ meniscus fiber optic probe data). IHC images of human repair cartilage were used to assess the repair cartilage PLS model prediction result for regions composed of hyaline (type II collagen with greater proteoglycan) and fibrocartilage (type I collagen). In meniscus, IHC images and Alcian blue staining were used as standard methods for assessment of type I collagen and proteoglycan distribution, respectively.

### Multivariate Analysis (Cluster analysis)

Cluster analysis of infrared imaging spectra has previously been performed to qualitatively differentiate tissue types [24]. With this method, regions of IR images are separated into two or more classes, or “clusters”, with similar spectral properties. A supervised cluster analysis was performed in this study, where four libraries of infrared spectra were created from spectra obtained from 1. FT-IRIS images of bone (primarily type I collagen), 2. FT-IRIS images of regions of cartilage repair tissue that were predominantly type I collagen, 3. FT-IRIS images of regions of cartilage repair tissue that were predominantly type II collagen, and 4. KBr pellets of pure aggrecan. The FT-IRIS spectral libraries were obtained from one tissue section of an ACI repair biopsy. IR spectra of type I and II collagen-rich regions from ACI repair tissue were chosen to create the library, instead of using pure component spectra, to assess the ability of the cluster analysis technique to differentiate fibrocartilage-like type I collagen from bone type I collagen. The libraries were then used to predict a distribution map of these tissue types in independent samples of repair cartilage (n = 3). Cluster analysis was performed using Fuzzy C-means, with four initial centroids and a Fuzzy index equal to 3.3. All data analysis were performed using ISys v5.0 (Malvern Instrument, Columbia, MD), and Unscrambler v10 (Camo, Norway).

## Results

### PLS models

#### Pure Components

**Figure 1:**
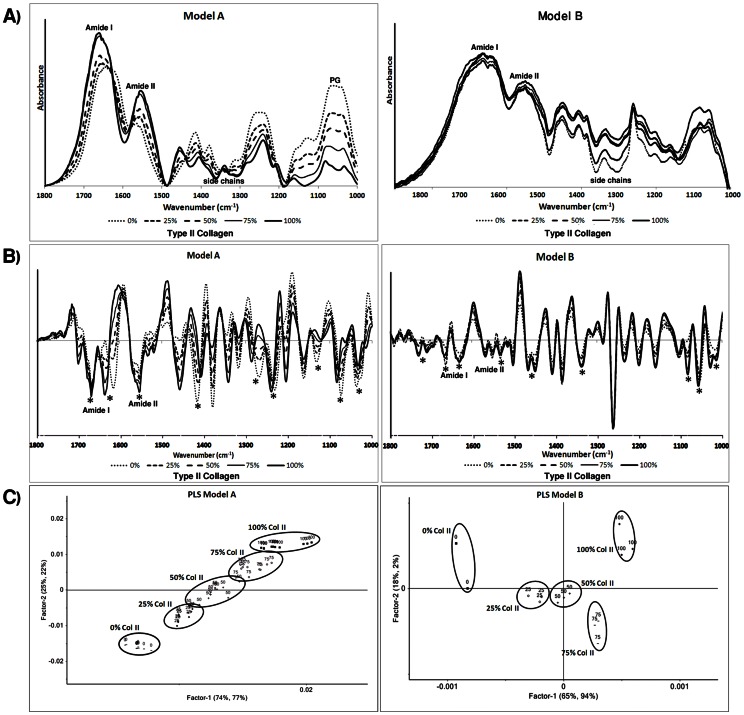
PLS score plots of pure component mixtures. Baselined infrared spectra (A), 2^nd^ derivative spectra (B) and PLS score plots of pure component mixtures in KBr pellets for Model A (mixtures of type II collagen and aggrecan) and Model B (mixtures of type I and type II collagen). Progressive changes in IR spectra with type II collagen variation can be seen. Asterisks in Figure 1B denote regions where second derivative spectra do not overlap. Mixtures with different compositions are completely distinguished in the PLS models.

IR spectra of pure component KBr mixtures (Models A and B) with varying type II collagen content show progressive changes in height and area under the amide I (1718–1594 cm^−1^), amide II (1594–1492 cm^−1^) and PG (1140-985 cm^−1^) absorbance bands (Figure 1A). Based on the broadening of the absorbance bands, it appeared that scattering was evident in the spectra of the type I and type II collagen mixtures, possibly due to incomplete grinding of the samples. Second derivative and MSC spectral analysis reduced the scattering artifact, and revealed more details on peak position (Figure 1B). Although the type II collagen second derivative spectra in models A and B (solid dark black lines) were not identical based on relative peak heights, the peak positions were essentially equivalent between the two models. There were small differences in several second derivative peak positions (ranging from 4 to 8 cm^−1^) of the type I and type II collagen spectra (Figure 1B), including in the amide I and II regions, in the side chain absorbance regions (near ∼1400 and 1300 cm^−1^), and in the glycosylation regions near 1100–1000 cm^−1^, but for the most part, significant overlap in the spectral frequencies precluded unique association of any peak position with a collagen type. This clearly motivated a multivariate analysis strategy for differentiation of these collagen types in tissues. Differences among second derivative spectra for the collagen and aggrecan mixtures were more obvious across the entire frequency range.

Both PLS models showed a complete discrimination among KBr pellet samples with varying composition (Figure 1C). However, Model A showed discrimination of percentage of type II collagen in both Factor 1 and Factor 2, while Model B showed discrimination of percentages of type II collagen based on Factor 1. Therefore, when using Model B for assessment of the amounts of type I and II collagen, only Factor 1 would be considered. PLS analysis parameters, including percentage of variation of the model explained by each factor, are summarized in Table 3.

**Table 3:** Parameters of PLS models used to predict component concentration.

	R-squared	RMSE (%)	% of variation explained by Factor 1 (variables)	% of variation explained by Factor 1 (response)	% of variation explained by Factor 2 (variables)	% of variation explained by Factor 2 (response)
**Model A**	0.97	4.36	74%	77%	25%	22%
**Model B**	0.98	4.88	65%	94%	18%	2%

#### Bone and Tendon Composition

Using Model B, FT-IRIS images of cortical bone and tendon both were predicted to have a fairly uniform distribution of type I collagen, with approximately 85% type I collagen (dry weight) in each tissue (Figure S1A and S1B). Although both tissues are considered to have no type II collagen, just under 5% of type II collagen was predicted in both tissues. Given that the RMSE of the models is ∼5%, this is within expected limits.

### Normal Articular Cartilage Composition

**Figure 2:**
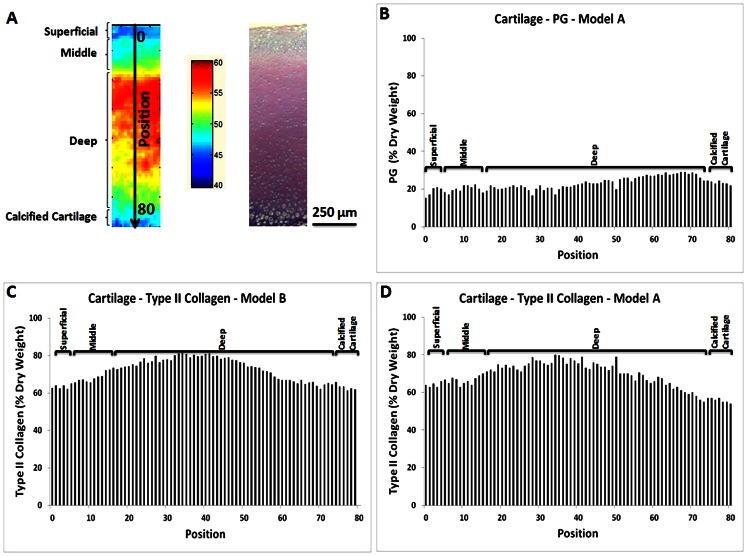
Articular cartilage matrix component concentration profile predicted using PLS models. Total protein amide I FT-IR image, where the color bar indicates the relative protein concentration as lower concentration (blue) through higher concentration (red), and Alcian blue histology image of articular cartilage (A). FT-IRIS data were collected from the articular surface to the subchondral bone, with the positions of data collection indicated on the IR image. Each position corresponds to a 25 micron region of data collection. PG (Panel B) and type II collagen (Panels C, D) concentration profiles in normal articular cartilage predicted using PLS models A (Panel D) and B (Panel C) (representative data shown for one sample). The Alcian blue-stained histology image shows the qualitative PG distribution, which is slightly higher in the deep zone, and similar to that predicted using Model A. Models A and B predicted similar distributions of type II collagen, where the deep zone contains a greater amount compared to the superficial and middle zones.

The superficial, middle, deep, and calcified cartilage zone were visualized in the FT-IRIS images from articular cartilage based on total protein (Amide I) (Figure 2A). This is the expected normal zonal structure of articular cartilage. The PLS model prediction showed that type II collagen and PG content varied through the thickness of the cartilage, with PG concentration increasing from the superficial to middle and deep zones (Figure 2B). The deep zone contained the highest type II collagen concentration, ∼80% dry weight, and the calcified cartilage zone contained the lowest type II collagen concentration, ∼55%. Similar concentration profiles from the superficial to calcified zone were found using models A and B (Figure 2C, 2D).

#### Human Cartilage Repair Tissue Composition

**Figure 3:**
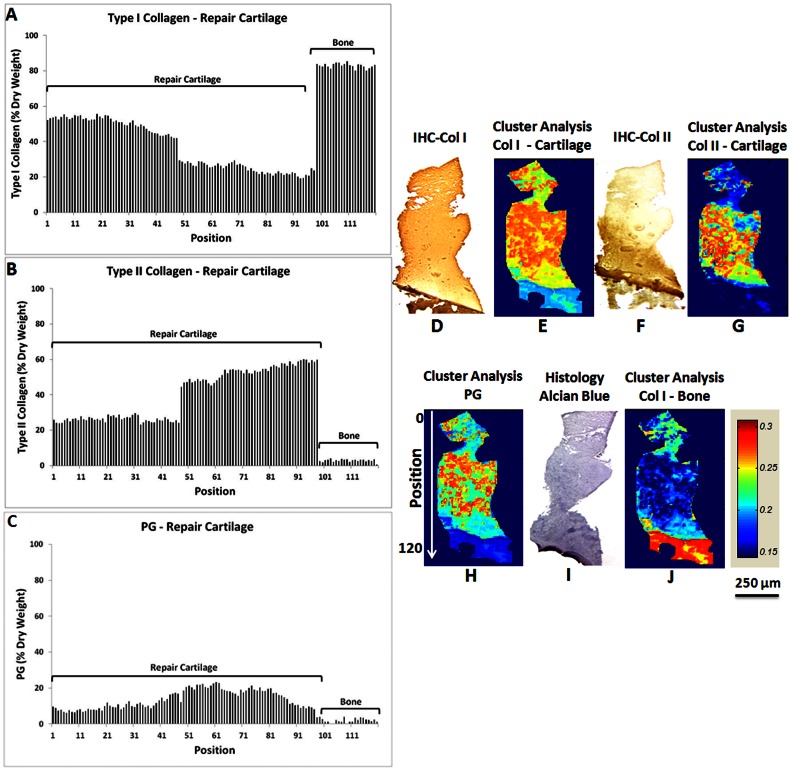
Repair cartilage matrix component concentration profile predicted using PLS models. Type I collagen (A), type II collagen (B), and PG (C) content of repair cartilage predicted by the PLS models show greater type II collagen is found in regions adjacent to bone (representative data shown for one sample). Each position corresponds to a 25 micron region of data collection. Cluster analysis-derived IR images show type I collagen (from repair cartilage spectral library), (E), type II collagen (from repair cartilage spectral library), (G), PG (from aggrecan spectral library), (H), and type I collagen (from bone spectral library), (J), distribution in repair cartilage and subchondral bone. IHC images of type I (D) and II (F) collagen and Alcian blue histology image (I) are used for comparison. Cluster analysis was done using Fuzzy C-means with four centroids defined. Higher values on the cluster analysis images scale show a closer distance to the corresponding centroid (tissue type). In the type II collagen IHC image, darker areas shows regions with more type II collagen (hyaline cartilage) and lighter areas indicate the presence of fibrocartilage-like tissue.

Excellent agreement was seen between type I collagen, type II collagen and PG content predicted in the FT-IRIS images by the PLS models, and the component distributions as visualized in the IHC and histology images (Figure 3A, B, C, D, F, I). Less than 5% type II collagen was predicted in subchondral bone, likely due to error in the model (Figure 3B). Similar to the PLS results of this tissue section, cluster analysis on the FT-IRIS image showed greater hyaline cartilage (type II collagen rich) in deeper regions of repair tissue, while type I collagen rich matrix was found throughout the repair tissue, but not in the bone (Figure 3E, G, H, J). However, there were some differences between the type I collagen and type II collagen distribution maps in the FT-IRIS images derived from cluster analysis, and the corresponding IHC images. This is likely due to the fact that the library of spectra utilized for the supervised cluster analysis were not necessarily pure components of type I or type II collagen, but were obtained from regions in FT-IRIS images that were primarily type I or type II collagen (as described in the Methods section). However, bone tissue type I collagen was only detected in the confirmed bone regions of the FT-IRIS images of the ACI biopsy, and not within the cartilaginous tissue. PG co-distributed primarily with the type II collagen rich regions, as expected.

#### Meniscus Composition

**Figure 4:**
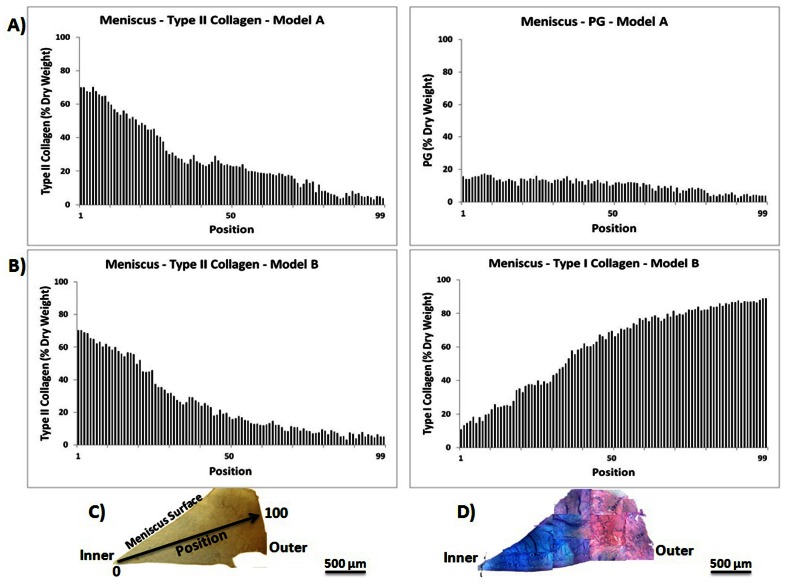
Meniscus histological section component concentration profile predicted using PLS models. Meniscus matrix composition prediction: FT-IRIS data obtained from a histological section, and predicted using PLS Model A (A) and B (B) (representative data shown for one sample). The inner region shows more PG and type II collagen compared to the outer region. The IHC image of type I collagen (C), and the Alcian blue histology image (D) show type I collagen and PG distribution respectively, which are in agreement with the predicted type I collagen and PG concentration, respectively. Each position corresponds to a 25 micron region of data collection.

FT-IRIS Data: Both PLS models predicted that the inner region of the meniscus tissue section contained greater quantities of type II collagen compared to the outer region (Figure 4A and 4B). Conversely, less type I collagen was predicted in the inner region compared to the outer, which was confirmed by type I collagen IHC (Figure 4C). In addition, proteoglycan concentration was also predicted to be greater in the inner region relative to the outer regions. This was confirmed by the Alcian blue staining (Figure 4D).

**Figure 5:**
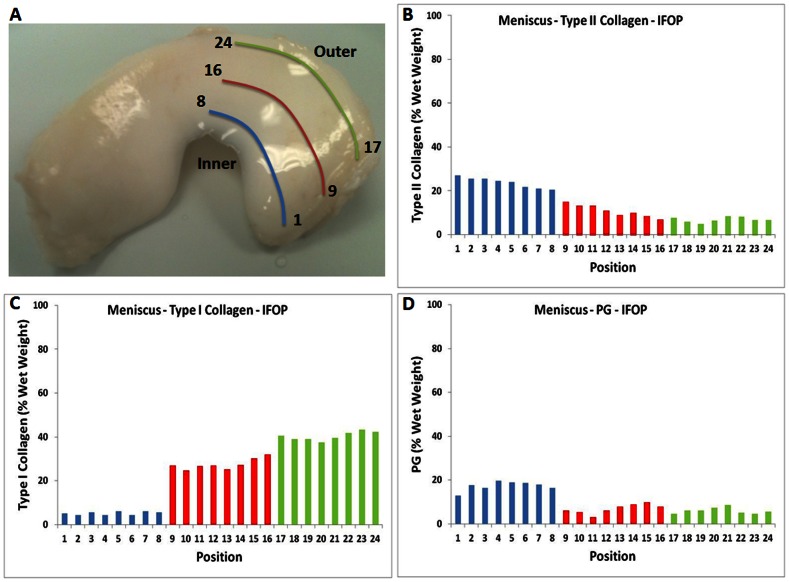
Intact meniscus component concentration profile predicted using PLS models. Type II collagen (A), PG (B) and type I collagen (C) content of lateral meniscus predicted using PLS analysis of IFOP spectra collected from the intact tissue (Model A used for panels A and B, and Model B used for panel C). Each position corresponds to a 1 mm diameter region of IFOP data collection. Similar to the results obtained for the analysis of meniscal tissue by IR imaging, the inner region was predicted to contain a higher concentration of type II collagen compared to the outer region. Each position corresponds to a 1 mm sampling region by IFOP.

IFOP Data: Analysis of the intact meniscus surface by IFOP showed a similar result to that obtained with the PLS analysis of the meniscus tissue sections (Figure 5), where the inner region surface was predicted to contain more type II collagen and PG compared to the outer region surface. There were small, non-linear fluctuations in the amount of these components observed from the medial to more lateral regions, e.g. among spectra 19–26, but the fluctuations were within the approximate error of prediction of the model, ∼5%, so it's not clear whether the variations are indeed meaningful. In these in situ surface studies, type II and type I collagen content was found to be relatively lower compared to what was found in the meniscus tissue sections. This is likely due to the fact that the intact tissue is sampled wet, and thus we obtain a wet weight percentage, as opposed to the dry weight percentage obtained from the dehydrated tissue sections. However, the predicted PG content was not noticeably lower for the surface IFOP measurements (wet weight) compared to the FT-IRIS image data from the thin section (dry weight). Thus, it is possible that the PG content on the meniscus surface is indeed different from that sampled from the center of the meniscus in the FT-IRIS image.

## Discussion

The current study demonstrates the ability to discriminate collagen types I and II based on spectral data for the first time. Comparison of spectral results with immunohistochemical data, the gold standard for differentiation of collagen types in tissue sections, confirms that this spectral approach can be used for semi-quantitative assessment of tissues with mixed collagen types. Further, IFOP data obtained from intact meniscus also discriminated regions of type I and type II collagen in that tissue.

Assessment of collagen types in histological tissue sections is most commonly done using IHC techniques. There have been many studies over the last 30 years that have performed IHC for evaluation of the types of collagen present in connective tissues, but of particular interest is the distribution of collagen types in cartilage repair tissue as a correlate to the quality of the tissue, as shown in [6], [7], [42]. However, immunohistochemical analysis has several limitations, including the dependence of the outcomes on the experience of the investigator [43]. It was also shown that choice of antibody panel and the interpretation of the reaction patterns are important factors in the ability to obtain reliable results and improve the sensitivity and specificity of the reaction [44]. These limitations motivate our studies to develop an alternative method to distinguish collagen types which would be a more objective and less bias-prone approach.

The spectral similarities between type I and type II collagen preclude evaluation of these collagen types based on one single spectral absorbance. Even though subtle differences among spectra were observed at several frequencies by evaluation of second derivatives, it was not possible to attribute these differences to specific features of the collagen molecules. Shifts in absorbance frequencies can occur if collagen molecules are in different hydration environments, or bound to different non-collagenous proteins, as is the case for type I and type II collagen. Thus, multivariate analyses, where many frequencies are utilized, have been explored. Several studies have shown that processing of FT-IR spectra using multivariate analysis methods provides quantitative information related to cartilage and bone tissue quality. Potter et al. utilized Euclidean distance analysis of IR imaging spectra obtained from histological sections to map collagen and chondroitin sulfate content of bovine nasal cartilage and engineered cartilage based on comparison to IR spectra of pure components [31]. Euclidean distance analysis has also been performed on IR spectra obtained from different collagen types in pure proteins [45], histological sections of human [32] and steer [27] cartilage to assess the relative concentration of collagen or PG in those tissues. Rieppo et al. performed cluster analysis to differentiate porcine cartilage repair tissue from normal articular cartilage [24], and Kobrina et al used this technique to differentiate histological zones in intact articular cartilage [46]. PCA analysis of FT-IR spectra was used by Yin et al to evaluate collagen and proteoglycan content of articular cartilage [47]. PLS analysis has been performed to assess the matrix constituents of engineered cartilage [33], proteoglycan content of articular cartilage [48], and cartilage degeneration using a fiber optic probe [21]. In the aforementioned studies, although the multivariate analyses permitted semi-quantitative assessment of differences in proteoglycan, specific analysis of type II collagen in tissues with other collagen types (e.g. repair cartilage) was not performed.

There are several potential sources of error in the current studies, including technical errors, such as Beer's law non-linearity, instrumental misalignment, inaccurate weighing of the pure component samples, and scattering in the KBr pellets [41], which may have contributed to broadening of the peak positions in the Model B spectra. However, similar results for prediction of type II collagen in tissues were obtained with both Models A and B, indicating that the spectral artifacts did not interfere significantly with the analyses. Errors could also arise from ignoring the contribution of small quantities of non-collagen or non-proteoglycan matrix component (such as glycolipids or glycoproteins) and cells, and using aggrecan with no link protein and hyaluronic acid as representative of proteoglycan in all tissues. In addition, the structure of the type I and II collagen powders used in the standard pellets is different from that of native collagen found in biological tissues with respect to lack of interactions with other matrix components, and possible differences in crosslinking, which can also result in errors in the models developed. Nonetheless our results showed that IR spectra collected from type I and II collagen pellets are similar enough to IR spectra of bone/tendon (type I collagen) and normal hyaline cartilage (type II collagen), respectively, for prediction in multivariate models. In the cluster analysis, the IR spectral library was generated based on type I and II collagen of ACI cartilage, as opposed to pure component spectra. The regions where the type I and type II collagen spectra were extracted from were chosen based on IHC identification of collagen type. However, as previously discussed, IHC analysis also has limitations regarding specificity. Further, the regions chosen for extraction of type I and type II collagen spectra also no doubt had other components present in small quantities, which would contribute to the errors in the models developed.

The variation in type II collagen content predicted by both models for normal cartilage was in a good agreement with the IR-derived collagen distribution shown in Figure 2A. Collagen content reached a maximum of ∼80% in the deep zone, which is also in agreement with the reported approximate relative amount of dry weight collagen concentration in articular cartilage [4]. The type II collagen quantity was at a minimum in the calcified cartilage zone, which could reflect the presence of type X collagen as the primary protein in this region [49], although we did not include type X collagen measurements in the current study. The predicted amount of proteoglycan (aggrecan) and type II collagen combined was ∼90% of the matrix dry weight throughout the superficial to deep zones which is also similar to what has been reported previously [1]. The type II collagen quantity measured using Models A and B, which contained different sources of type II collagen, resulted in similar compositional variations throughout the tissue depth. It is important to note that these measurements are semi-quantitative, reflecting percentage of a type of collagen, and not a fully quantitative assessment. However, these data confirm that the methods developed are not sensitive to collagen source.

A positive clinical outcome in ACI procedures has been correlated with the amount of hyaline repair tissue [10], [50], [51], whereas the presence of fibrocartilage with little hyaline cartilage has been linked to failure of the treatment [51]. In our previous study, we demonstrated that FT-IRIS data obtained from ACI biopsies were equivalent to histologic or IHC parameters for prediction of clinical outcome based on the Lysholm score [10]. The results from the current study augment those findings by specifically assessing collagen type with FT-IR, and thus provide another non-destructive, measure of tissue quality. Potential clinical application was further shown by assessment of collagen type and PG distribution in bovine meniscus in both imaging data and in fiber optic data. Results were in agreement with previous immunostaining studies, including evaluation of rabbit [52] and human meniscus [53], where it was confirmed that type II collagen is primarily localized at the interior site of the medial meniscus. Notwithstanding these positive results, a limitation of the mid-infrared fiber optic studies is that penetration is restricted to ∼10 microns below the tissue surface, and thus only superficial components are evaluated. In the current study, however, this sampling modality was sufficiently sensitive for evaluation of the distribution of matrix components in the intact tissue, as it was shown in our previous study [54].

Although mid-infrared fiber optic spectroscopic analysis has not yet been used clinically for evaluation of cartilage repair tissue or meniscus, it has been used to assess cartilage degeneration [21], [33]. The application of a mid-infrared fiber optic probe to evaluate degenerative cartilage in harvested human tissues was shown in earlier studies from our lab, [21], [54], where a PLS analysis method was used to correlate spectral data acquired from an IFOP to the visual score [21], and histological score [54] for degenerative cartilage. We showed that the PLS model developed based on infrared fiber optic probe spectroscopy data was able to predict the histological grade of degenerative tissue comparably to histological Mankin score, the gold standard of such analysis. In addition, Bi et al. evaluated disease progression in an OA rabbit model, using a PLS model based on IFOP data obtained from femoral cartilage [55].

Nevertheless, there would be some limitations in translation of this methodology to predict collagen type in a clinical application. Since the current configuration of the ATR probe crystal has a 1 mm diameter, this is the smallest tissue region that can be evaluated. While this still may be sufficient for assessment of repair of a tissue lesion, newer fiber optic configurations are being developed with smaller sampling capability. Our previous study demonstrated that data collected from cartilage surface reflects the tissue quality and degree of degeneration [54], which could be useful in clinical procedures where definition of the margins of a lesion are required. However, it remains to be confirmed whether this is the case for repair tissue as well, and whether surface measurements reflect full depth tissue quality. Another potential limitation during in vivo assessment is interference from absorbances present in synovial fluid and blood. These may overlap with absorbances from the proteins in cartilage, and have to be taken into account with development of multivariate models. Although the current studies performed did not address these potential concerns in data analysis, future studies will focus on these issues.

Together, the results of the current and earlier studies lay the foundation for in situ evaluation of cartilage repair tissue and meniscus repair using minimally-invasive infrared spectroscopy. The use of an IR probe to evaluate tissue composition arthroscopically for determination of quality without the need for tissue harvest could significantly augment clinical management of degenerative cartilage diseases and efficacy of related therapeutic interventions.

## Supporting Information

Figure S1 **Type I and type II collagen concentration profile in bone (Panel A) and tendon (Panel B) predicted using PLS model B (representative data shown for one sample).** The positions of FT-IRIS data collection are indicated on the IR image. Each position corresponds to a 25 micron region of data collection.
(TIF)
