# Supplementary figures and images for: Fourier Transform Infrared Imaging and Infrared Fiber Optic Probe Spectroscopy Identify Collagen Type in Connective Tissues
Source: PLoS One. 2013 May 22;8(5):e64822. doi: 10.1371/journal.pone.0064822 (PMC3661544; doi:10.1371/journal.pone.0064822)

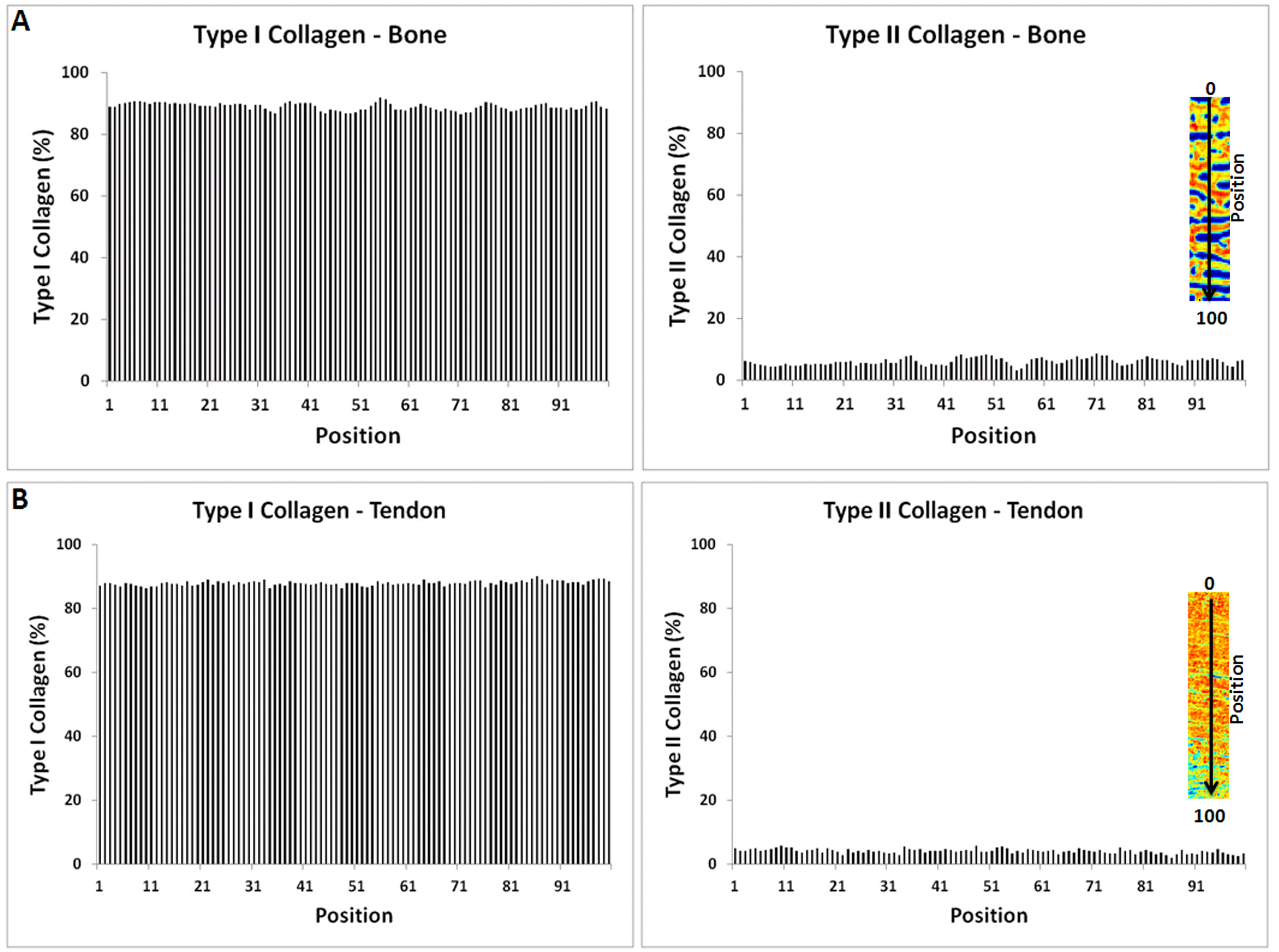

Supplement: Figure S1 — Type I and type II collagen concentration profile in bone (Panel A) and tendon (Panel B) predicted using PLS model B (representative data shown for one sample). The positions of FT-IRIS data collection are indicated on the IR image. Each position corresponds to a 25 micron region of data collection. (TIF) [file pone.0064822.s001.tif]
